# Maternal Inulin Supplementation Alters Hepatic DNA Methylation Profile and Improves Glucose Metabolism in Offspring Mice

**DOI:** 10.3389/fphys.2020.00070

**Published:** 2020-02-07

**Authors:** Qian Zhang, Xinhua Xiao, Jia Zheng, Ming Li, Miao Yu, Fan Ping, Tong Wang, Xiaojing Wang

**Affiliations:** Department of Endocrinology, Key Laboratory of Endocrinology, Ministry of Health, Peking Union Medical College Hospital, Peking Union Medical College, Chinese Academy of Medical Sciences, Beijing, China

**Keywords:** prebiotics, methylation, epigenetics, high fat diet, nutrition *in utero*

## Abstract

**Scope:**

As a prebiotic, inulin may have a protective effect on glucose metabolism. However, the mechanism of inulin treatment on glucose intolerance in offspring exposed to a maternal high-fat (HF) diet is still not clear. Here, we examined the hepatic DNA methylation profile to determine how maternal inulin supplementation modified glucose metabolism in offspring mice.

**Procedures:**

Female mice were fed a HF diet, control diet (CON), or a HF diet with inulin supplementation (HF-inulin) during gestation and lactation. Upon weaning, pup livers were obtained. A hepatic genome DNA methylation array was performed.

**Results:**

Pups exposed to a maternal HF diet exhibited glucose intolerance and insulin resistance. Maternal inulin treatment moderated glucose metabolism. A DNA methylation array identified differentially methylated regions associated with 970 annotated genes from pups exposed to a HF diet in response to maternal inulin treatment. In particular, the wingless-type MMTV integration site family member 5A (*Wnt5a*) gene was hypermethylated, and the phosphatidylinositol-4-phosphate 3-kinase catalytic subunit type 2 alpha (*Pik3c2a*), phosphatidylinositol-4-phosphate 3-kinase catalytic subunit type 2 beta (*Pik3c2b*), and phosphoinositide-3-kinase regulatory subunit 2 (*Pik3r2*) genes were hypomethylated in inulin-treated pups. Consistently, hepatic *Wnt5a* gene expression was reduced and *Pik3c2a*, *Pik3c2b*, and *Pik3r2* gene expression were increased in the inulin group.

**Conclusion:**

Maternal inulin treatment improved glucose intolerance by changing DNA methylation and gene expression of *Wnt5a* and *Pi3k* in mice exposed to a maternal HF diet.

## Introduction

Diabetes and its complications have become a major cause of death worldwide. Type 2 diabetes (T2D) results in great health and social burdens both in developing and developed countries. The T2D population has grown rapidly in recent years. Approximately 425,000 patients are diagnosed with T2D each year. In total, the global population of patients affected by T2D has already reached 415 million. Moreover, this number is estimated to reach 642 million in 2045 (Jaacks et al., 2016). Researchers have tried to explain the reason for the sharp increase in T2D. Genetic factors are important for the incidence of T2D. However, these factors cannot explain the incidence entirely. Environmental and lifestyle conditions also contribute substantially to this large increase in the rate of T2D (Dendup et al., 2018). In addition to adult life environmental factors (lifestyle), increasing evidence has shown that early-life living conditions also result in the incidence of metabolic disease (Vaiserman, 2017; Estampador and Franks, 2018; Zimmet et al., 2018). Research results from both monozygotic twin studies (Yajnik, 2013) and Pima Indians (Dabelea et al., 2000) have validated this idea.

Increasing evidence indicates that epigenetic regulation plays an important role in the linkage between the early environment and the incidence of metabolic diseases in later life (Bianco-Miotto et al., 2017). Abundant studies support epigenetic change as an important factor in the occurrence and development of metabolic diseases, such as obesity and T2D (Bansal and Simmons, 2018; Cheng et al., 2018). In 1992, Professors Hales and Barker raised the “thrifty phenotype” hypothesis (Hales and Barker, 1992). Until now, accumulating evidence in human and animal experiments have supported this hypothesis (Ravelli et al., 1976; Begum et al., 2012). Recent ideas have focused on the idea that over-nutrition and under-nutrition in early life cause major tissue and organ dysfunction in the fetal period and lead to metabolic dysfunction in adults, including effects on pancreatic β cells and adipose tissue (Godfrey and Barker, 1995; Tarry-Adkins and Ozanne, 2011). Both low birth weight (LBW, <2,500 g) and high birth weight (HBW, >4,000 g) will increase the risk of T2D in adults (Palatianou et al., 2014).

In recent years, scientists have found that epigenetic changes play a central role in the mechanism of early programing of metabolic diseases (Vaiserman et al., 2009; Davegardh et al., 2018; Szabo et al., 2018). Through developmental and differential processes, the epigenome may change dramatically. Epigenetic changes usually constitute DNA methylation, histone modification, and microRNA (miRNA) profile changes. DNA methylation is a phenomenon of the addition of a methyl group at the 5th position of the cytosine ring (Elhamamsy, 2016). Methylation in gene promoter regions usually inhibits gene transcription (Yin et al., 2017).

Prebiotics selectively stimulate the growth and/or activity of beneficial bacteria and have positive effects on the host gut tract. Prebiotics can reduce inflammation, improve gut permeability, and stimulate the growth of beneficial bacteria, including *Bifidobacterium* and *Lactobacillus*, in mice and human subjects (Roberfroid and Delzenne, 1998; Cani et al., 2009).

As a prebiotic, inulin is naturally present in a large variety of plants, including chicory. Inulin extracted from chicory includes a series of fructose molecules, which have 2–60-unit degrees of polymerization (DPs). Inulin is not digested in the human gastrointestinal system, but is fermented by gut bacteria. Through fermentation, the end-products are lactate and short-chain fatty acids (SCFAs), including acetate (Bornet, 1994; McBain and Macfarlane, 1997). Both human and animal experiments have shown that inulin has beneficial effects on metabolism, such as inhibiting increases in body weight and fat mass, improving blood glucose control, reducing inflammation, and increasing the abundance of *Bifidobacteria* in the gut (Delzenne et al., 2013). Moreover, inulin can increase satiety (Cani et al., 2005; Cani et al., 2006a) and increase glucagon-like peptide-1 (GLP-1) (Cani et al., 2006b), which stimulates insulin secretion after an oral glucose load, activates pancreatic β-cell proliferation, and inhibits pancreatic β-cell apoptosis (Meier and Nauck, 2005). In prediabetic subjects (Guess et al., 2016), individuals with type 1 diabetes (T1D) (Ho et al., 2016) and T2D patients (Liu et al., 2017), inulin-type fructans have been shown to moderate glucose intolerance.

Recently, possible mechanisms involving the gut microbiota and epigenetic moderation in human metabolic disease have been addressed (Remely et al., 2015). The free fatty acid receptor 3 (FFAR3) gene is hypomethylated in obese and T2D patients; moreover, the abundance of *Faecalibacterium prausnitzii* was significantly reduced (Remely et al., 2014). In pregnant women, blood DNA methylation patterns are associated with gut microbiota profiles (Kumar et al., 2014). The Firmicutes:Bacteroidetes ratio and the abundance of lactic acid bacteria are higher in T2D patients than in lean controls, which is accompanied by hypomethylated levels of inflammatory molecules such as the Toll-like receptor 2 (*Tlr2*) and *Tlr4* genes (Remely et al., 2015). However, the mechanism of DNA methylation moderation in the effect of maternal prebiotic supplementation on pups is still lacking.

Liver is the major organ in glucose metabolism. There are numerous enzymes and metabolic reaction occurred in liver (Petersen et al., 2017). Herein, inulin was investigated to evaluate the effect of prebiotic supplementation *in utero* on DNA methylation status in pup livers. We hypothesized that maternal prebiotic supplementation would lead to beneficial DNA methylation shifts in pups exposed to an intrauterine high-fat (HF) diet.

## Materials and Methods

### Animal Treatments and Diets

**FIGURE 1:**
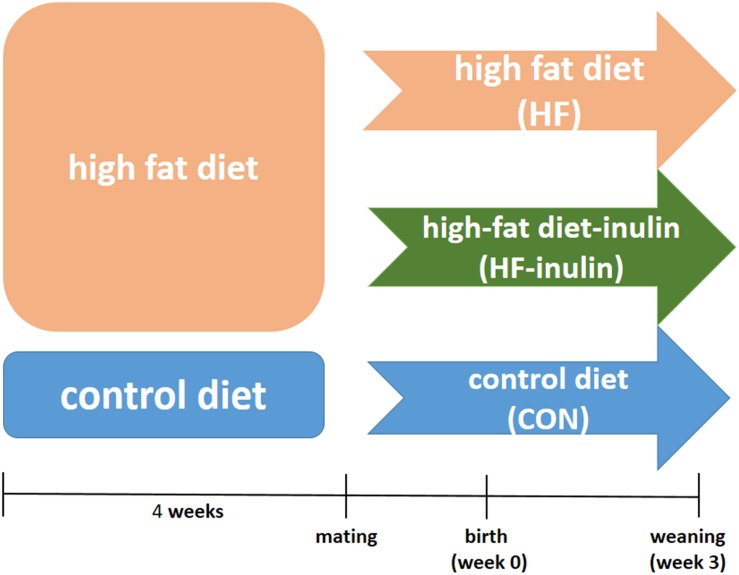
Animal experiment design.

All experiments were performed in accordance with the Guide for the Care and Use of Laboratory Animals, 8th ed., 2011, using protocols approved by the Animal Care Committee of Peking Union Medical Hospital (Permit Number: MC-07-6004). Five-week-old female C57BL6/J mice were housed under a constant 12-h light and dark cycle at an ambient temperature of 23°C with free access to food and water. A total of 30 female mice were randomly assigned to a control American Institute of Nutrition-93G (AIN-93G) diet (CON, *n* = 10; kcal%: 10% fat, 20% protein, and 70% carbohydrate; 3.85 kcal/gm; Research Diets, Inc.) (Reeves et al., 1993) or a HF diet (HF, *n* = 20; kcal%: 45% fat, 20% protein, and 35% carbohydrate; 4.73 kcal/gm; Research Diets, Inc.). The AIN-93G diets contain 50 g wood fiber/kg diet as the fiber source (Reeves, 1997). After 4 weeks, the mice were mated with males. The onset of pregnancy was determined by the presence of a vaginal plug. Pregnant mice in the HF group were randomly divided into the HF group (continued to be fed a HF diet, *n* = 10) or the HF-inulin group [HF diet with 10% wt/wt inulin supplement (Vilof^TM^ Soluble Dietary Fiber; BAHEAL Medical Inc., Qingdao, China and Fengning Ping’an High-tech Industrial Co., Ltd., Heber, China), *n* = 10]. For avoiding sex differences on the effect of maternal inulin treatment on glucose metabolism (Yokomizo et al., 2014), male pups were sacrificed by decapitation at weaning (3-week-old). The livers were immediately frozen in liquid nitrogen and stored at −70°C. Figure 1 shows the experimental protocol.

### Body Weight and Fasting Blood Glucose Analysis

Body weight was measured at 3 weeks of age in pups. Fasting blood glucose was also measured (Contour TS glucometer, Bayer, Hamburg, Germany).

### Oral Glucose Tolerance Test

After fasting for 12 h, an oral gavage of glucose (2.0 g/kg body weight) was given to each mouse. Blood glucose levels were measured before and after glucose loading at 15, 30, 60, and 120 min. The area under the curve (AUC) of the oral glucose tolerance test (OGTT) was calculated.

### Serum Biochemical Measurements

After 12 h of fasting, blood samples were collected from mice pups at 3 weeks of age. The serum fasting insulin levels were assayed using a mouse insulin enzyme-linked immunosorbent assay (ELISA) kit [Millipore, Billerica, MA, United States, in duplicates, coefficient of variability (CV) < 9%]. The Homeostasis Model of Insulin Resistance (HOMA-IR) index was calculated using the following formula: HOMA-IR = (fasting insulin × fasting glucose)/22.5.

### DNA Preparation and DNA Methylation Microarray

Genomic DNA was extracted from the livers of the offspring in the HF, and HF-inulin groups (*n* = 3 in each group) using a DNeasy Blood & Tissue Kit (Qiagen, Fremont, CA, United States). The integrity, purity, and concentration of each DNA sample were assessed on a NanaDrop ND-1000 Spectrophotometer (Thermo Fisher Scientific Inc., Waltham, MA, United States). Three micrograms of DNA were sonicated into 100–500 bp fragments by using a Bioruptor sonicator (Diagenode). One microgram of fragmented genomic DNA was immunoprecipitated with a mouse monoclonal anti-5-methylcytosine antibody (Diagenode). Then, 200 μL of anti-mouse IgG magnetic beads were used to recover the immunoprecipitated DNA fragments, which were incubated for an additional 2 h at 4°C with agitation. For DNA labeling, 1 μg of DNA from each sample was incubated for 10 min at 98°C with Cy5 [immunoprecipitated (MeDIP) samples] or Cy3 (input samples) primers. Labeled DNA was hybridized to the Arraystar Mouse ReqSeq Promoter Array (Arrarystar Inc., Rockville, MD, United States). This array is designed to investigate the epigenetic modifications and transcription factor binding sites within RefSeq Gene promoter regions, including 22,327 gene promoter regions. Finally, arrays were washed and scanned with an Agilent Scanner G2505C (Agilent Technologies, Waldbronn, Germany).

### Data Normalization and Analysis

From the normalized log2 ratio data, a sliding-window peak-finding algorithm provided by NimbleScan v2.5 (Roche NimbleGen Inc.) was applied to find the enriched peaks. NimbleScan detects peaks by searching for at least two probes above a *p*-value minimum cutoff (−log10) of 2 and maximum spacing of 500 bp between nearby probes within the peaks. To compare differentially enriched regions between the HF-inulin group and the HF group, the log2 ratios were averaged and then used to calculate *M*′ for each probe: *M*′ = Average (log2^MeDIP(HF–inulin)/Input(HF–inulin)^)−Average (log2^MeDIP(HF)/Input(HF)^). The NimbleScan sliding-window peak-finding algorithm was run on these data to find the differential enrichment peaks (DEPs). The DEPs, identified by the NimbleScan algorithm, were filtered according to the following criteria: (1) At least one of the two groups has a median log2 MeDIP/Input ≥ 0.3 and a median *M*′ > 0. (2) At least half of the probes in a peak may have a CV ≤ 0.8 in both groups. To separate strong CpG islands from weak CpG islands, promoters were categorized into three levels: high CpG promoters/regions (HCP, high CpG density promoter), intermediate CpG promoters/regions (ICP, intermediate CpG density promoter), and low CpG promoters/regions (LCP, low CpG density promoter) (Weber et al., 2007).

### Pathway and Bioinformatics Analysis of Array Results

Differentially methylated genes (DMGs) were annotated using Gene Ontology (GO) terms [biological process (BP), cellular component (CC), and molecular function (MF)] as well as KEGG pathway enrichment using DAVID Bioinformatics Resources 6.7^1^ (Dennis et al., 2003). An adjusted *P*-value < 0.01 for GO term analysis and adjusted *P*-value < 0.05 for KEGG pathway analysis after the Benjamini–Hochberg procedure is considered as significant difference.

### Bisulfite Sequencing PCR

Bisulfite sequencing PCR (BSP) primers were designed using Methyl Primer Expression software 1.0 (Applied Biosystems, Foster City, CA, United States), as shown in Table 1. Genomic DNA extractions of the three groups (*n* = 10 in each group) were performed using the same method mentioned above. One microgram of DNA samples was converted using an EZ DNA Methylation Kit (Zymo Research, Irvine, CA, United States). The converted DNA was then amplified by PCR. PCR products were purified using a QIAquick Gel Extraction Kit (QIAGEN) and ligated to the pMD18-T Vector (Takara, Shiga, Japan). The plasmids were then purified using the PureLink Miniprep Kit (Invitrogen, Thermo Scientific Inc., Waltham, MA, United States). A minimum of 10 clones from each mouse were sequenced on the ABI 3730 sequence. Sequence analysis was performed using QUMA (Kumaki et al., 2008).

**TABLE 1:** Primers for bisulfite sequencing.

**Gene**	**Accession number**	**Primer sequence (from 5′ to 3′)**	**Production size**	**CpG number**
*Wnt5a*	NM_009524	F: 5′-TATTTAGAGGTGTTTAGAAGTTTTGGAGTTTGGATTTTYGGTTTATTTAAAT-3′	276	6
		R: 5′-AACTACATTTACTAACACCTCTACAAAAAAAACCTCACTAACATAAATCCTA-3′		
*Pik3c2a*	NM_011083	F: 5′-TYGYGTTTAYGAGAAAGGTATGATTATTATGGGGTTTGGGTGTGATGTTTGGT-3′	296	22
		R: 5′-CTTACRATTACCTAATTTAAAATTTTAACCCCAAACCRCCCAAAAATTAACT-3′		
*Pik3c2b*	NM_001099276	F: 5′-GGAAAATGTTTGATATAGGTGTTTTAAGGGAGGTGTTYGGAGAAAATAATAT-3′	311	9
		R: 5′-AACAAATTATCRTCRTTTTCCAAACTAAAACCCCTTACTCTAAATCAAATAA-3′		
*Pik3r2*	NM_008841	F: 5′-GTAATTTATTGAATTTGGATTTTGTGTAAGAGTAGTGAGTATGTTTAATTGTY-3′	419	31
		R: 5′-AAAAAACCRAACRACCTCAACTCCAAACCTTAAAAATTAACTCRAAAACCRC-3′		

**Wnt5a*, wingless-type MMTV integration site family, member 5A; *Pik3c2a*, phosphatidylinositol-4-phosphate-3-kinase catalytic subunit type 2 alpha; *Pik3c2b*, phosphatidylinositol-4-phosphate-3-kinase catalytic subunit type 2 beta; *Pik3r2*, phosphoinositide-3-kinase regulatory subunit 2.*

### RNA Isolation and Quantitative Real Time-PCR Analysis

Hepatic RNA from three groups (*n* = 10 in each group) was extracted using an RNeasy Mini Kit (Qiagen, Germantown, MD, United States), and cDNA was synthesized using oligo-dT and random primers (TaKaRa, Shiga, Japan). Quantitative real time-PCR (qPCR) was performed using a SYBR green real-time PCR master mix (Applied Biosystems, Foster City, CA, United States) on the ABI 7900 detection system (Applied Biosystems, Foster City, CA, United States). *Gapdh* was used as the internal control. Primers are listed in Table 2.

**TABLE 2:** Primers for qPCR.

**Gene**	**Accession**	**Primer sequences**	**Production**
	**number**	**(from 5′ to 3′)**	**size**
*Wnt5a*	NM_009524	F: 5′-GTTGCTCCGGCCCAGAAG-3′	112
		R: 5′-AGAAAAACGTGGCCAAAGCC-3′	
*Pik3c2a*	NM_011083	F: 5′-CAGTCGAAGCTCTCCTCAGC-3′	132
		R: 5′-AAATACCAGGACCTCACGCT-3′	
*Pik3c2b*	NM_001099276	F: 5′-GACTAGGCGATTCGGCGTTG-3′	146
		R: 5′-TGAGACAATAGCGCGAACGG-3′	
*Pik3r2*	NM_008841	F: 5′-TGGAGTTCCTAGGACCCGTG-3′	113
		R: 5′-TGGGAGTATGTGGCCTGACT-3′	

**Wnt5a*, wingless-type MMTV integration site family, member 5A; *Pik3c2a*, phosphatidylinositol-4-phosphate-3-kinase catalytic subunit type 2 alpha; *Pik3c2b*, phosphatidylinositol-4-phosphate-3-kinase catalytic subunit type 2 beta; *Pik3r2*, phosphoinositide-3-kinase regulatory subunit 2.*

### Statistical Analysis

Prism 5.0 (GraphPad Software Inc., San Diego, CA, United States) was used for all statistical analyses. Data are presented as the mean ± SEM. Comparisons between two groups were performed using Student’s *t*-tests. One-way ANOVA was used to detect differences among comparison groups followed by Tukey’s *post hoc* test for comparing groups. *P* < 0.05 was considered statistically significant.

## Results

### Maternal Inulin Supplementation Ameliorated Body Weight, Blood Glucose, and Insulin Resistance in Offspring

**FIGURE 2:**
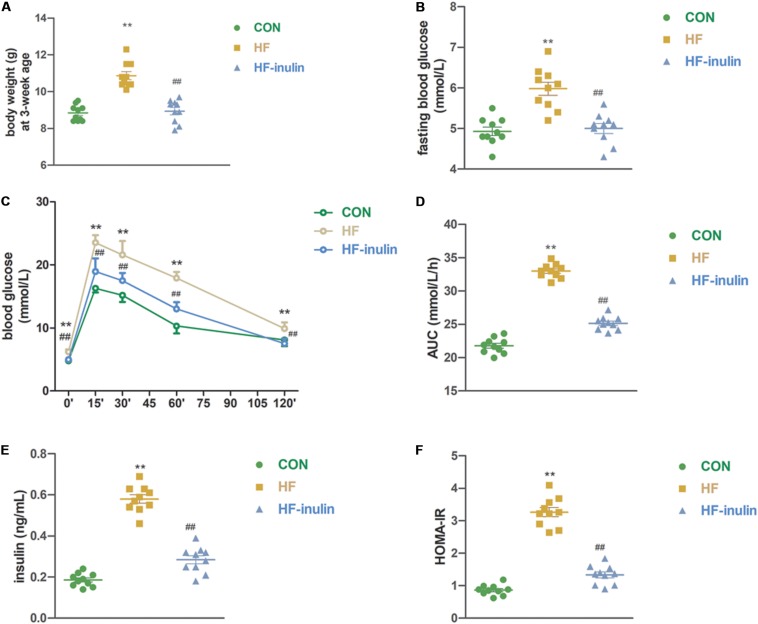
The effect of maternal early inulin treatment on metabolic variables of male mice offspring. **(A)** Body weight at weaning; **(B)** fasting blood glucose; **(C)** oral glucose tolerance test (OGTT); **(D)** area under curve (AUC) in OGTT; **(E)** serum insulin; **(F)** HOMA-IR. **P < 0.01 vs. CON group; ^##^*P* < 0.01 vs. HF group. Values are mean ± SEM (*n* = 10). CON, control diet; HF, high-fat diet; HF-inulin, high-fat diet with 10% wt/wt inulin supplement.

Pups from HF dams exhibited a 22.5% higher bodyweight at weaning (*P* < 0.01, Figure 2A). Inulin supplementation decreased body weight at weaning (*P* < 0.01, Figure 2A). Both serum fasting glucose levels and glucose levels from the OGTT increased significantly in the HF group pups (*P* < 0.01, Figures 2B,C). The AUC of the OGTT increased by 49.7% (*P* < 0.01, Figure 2D). Compared with the HF group, fasting blood glucose concentrations were lower in the HF-inulin group pups (*P* < 0.01, Figure 2B). Serum insulin levels were higher in the HF group pups than in the CON pups at 3 weeks of age (*P* < 0.01, Figure 2E). Glucose tolerance and insulin resistance tests revealed decreased glucose tolerance (*P* < 0.01, Figures 2C,D) and increased insulin resistance (*P* < 0.01, Figure 2F) in the HF group pups, respectively. Inulin supplementation ameliorated glucose tolerance (*P* < 0.01, Figure 2D) and insulin resistance (*P* < 0.01, Figure 2F).

### Maternal Inulin Supplementation Affected Hepatic DNA Methylation in Offspring

**FIGURE 3:**
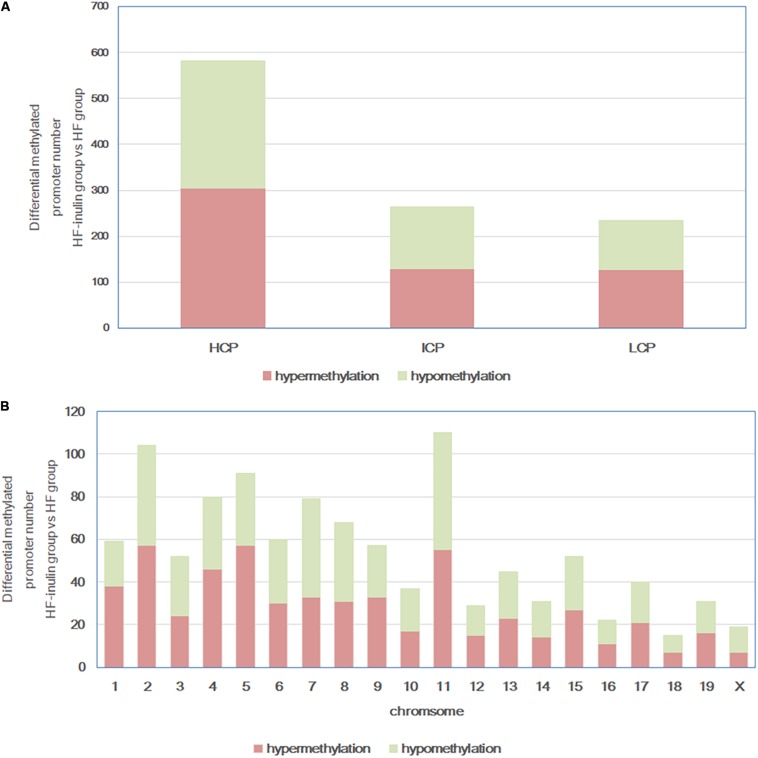
Differentially methylated promoters between HF-inulin group and HF group. **(A)** CpG density of differentially methylated promoters. **(B)** Chromosomal distribution of differentially methylated promoters. Red, differentially hypermethylated promoters; green, differentially hypomethylated promoters. Classification of all promoters with high (HCP, high CpG density promoter), intermediated (ICP, intermediate CpG density promoter), and low (LCP, low CpG density promoter) CpG content.

DNA methylation array data are available from the NCBI’s Gene Expression Omnibus repository^2^ (GEO) under the series accession number GSE136766. HF-inulin group and HF group hepatic DNA methylation status were compared. A total of 1081 DMRs (970 annotated genes) were identified in 20 chromosomes (Figure 3), particularly on chromosomes 2, 4, 5, 7, 8, and 11. Among these DMRs, 582 (53.84%) were located in HCP, 264 (24.42%) in ICP, and 235 (21.74%) in LCP (Figure 3A). Five hundred sixty-two hypermethylated DMRs (51.99%) and 519 hypomethylated DMRs (48.01%) were found in the HF-inulin group compared with the DMRs of the HF group (Figure 3B).

### Maternal Inulin Supplementation Affected Several Signaling Pathway in Offspring Liver

Differentially methylated genes were annotated with GO terms and Kyoto Encyclopedia of Genes and Genomes (KEGG) pathways. The top five significant BPs in GO terms were multicellular organism development, positive regulation of transcription from the RNA polymerase II promoter, dendrite development, negative regulation of protein localization to the cell surface, and regulation of multicellular organism growth (adjusted *P* < 0.01, Supplementary Table 1).

**FIGURE 4:**
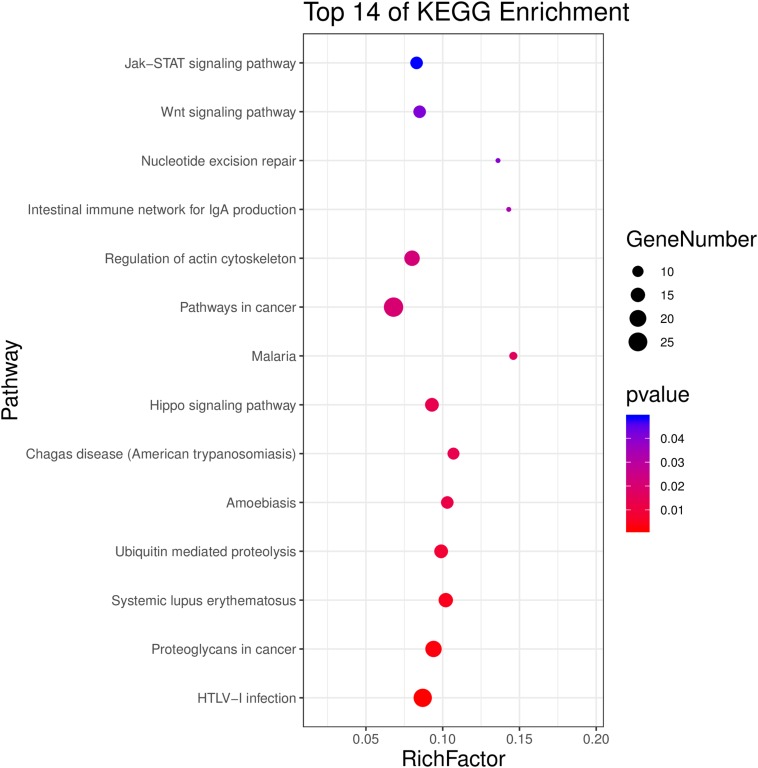
Top 14 significant KEGG pathways of differentially methylated genes between HF-inulin group and HF group (adjusted *P* < 0.05).

The investigation of KEGG pathways demonstrated that the top 14 significant pathways were HTLV-1 infection, proteoglycans in cancer, systemic lupus erythematosus, ubiquitin-mediated proteolysis, amoebiasis, Chagas disease, hippo signaling pathway, malaria, pathways in cancer, regulation of actin cytoskeleton, intestinal immune network for IgA production, nucleotide excision repair, WNT signaling pathway, and Jak-STAT signaling pathway (adjusted *P* < 0.05, Figure 4 and Supplementary Table 2).

### Maternal Inulin Supplementation Activated *Wnt5a* Methylation and Inhibited *Pik3c2a*, *Pik3c2b*, and *Pik3r2* Methylation in Offspring Livers

**FIGURE 5:**
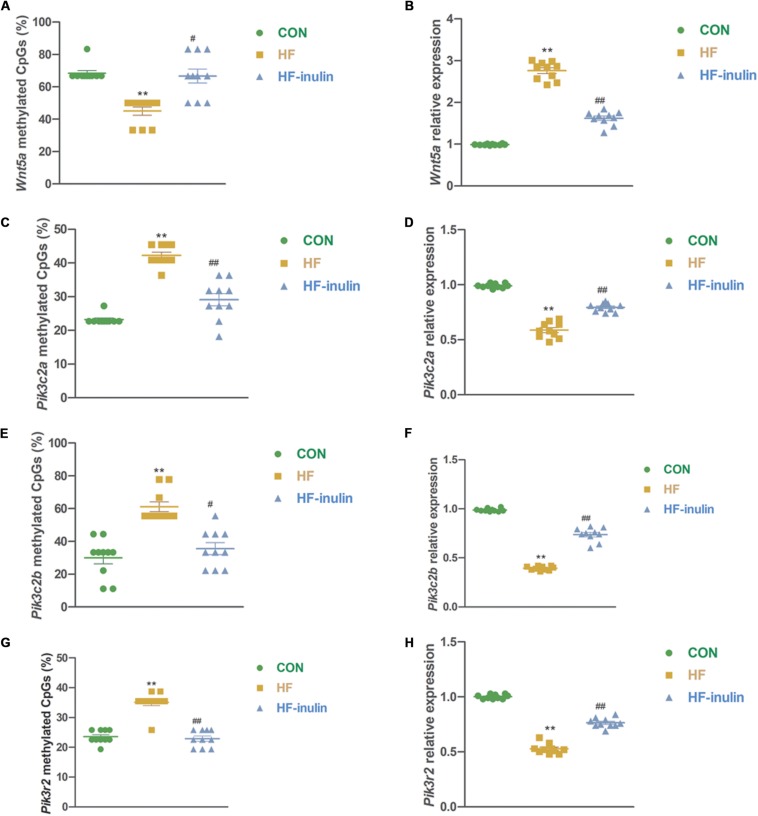
Validation of methylation array using bisulfite sequencing. Methylation ratio of *Wnt5a* **(A)**, *Pik3c2a* **(C)**, *Pik3c2b* **(E)**, and *Pik3r2* **(G)** in different groups and relative gene expression of *Wnt5a* **(B)**, *Pik3c2a* **(D)**, *Pik3c2b* **(F)**, and *Pik3r2* **(H)** in different groups. **P < 0.01 vs. CON; ^#^*P* < 0.05, ^##^*P* < 0.01 vs. HF. Values are mean ± SEM (*n* = 10). CON, control diet; HF, high-fat diet; HF-inulin, high-fat diet with 10% wt/wt inulin supplement.

To further validate the inulin supplementation-induced DNA methylation changes described above, four DMGs associated with the *Wnt* and *Pi3k* signaling pathway genes *Wnt5a*, *Pik3c2a*, *Pik3c2b*, and *Pik3r2* were selected for technical validation using independent bisulfite sequencing in three groups. Consistent with the methylation array results, the BSP results showed that *Wnt5a* gene methylation was decreased (*P* < 0.01, Figure 5A), while *Pik3c2a*, *Pik3c2b*, and *Pik3r2* gene methylation was increased in mice in the HF group (*P* < 0.01, Figures 5C,E,G). Inulin supplementation increased *Wnt5a* gene methylation (*P* < 0.05, Figure 5A) and reduced *Pik3c2a*, *Pik3c2b*, and *Pik3r2* gene methylation (*P* < 0.05 or *P* < 0.01, Figures 5C,E,G).

### Maternal Inulin Supplementation Changed *Wnt5a*, *Pik3c2a*, *Pik3c2b*, and *Pik3r2* Gene Expression in Offspring

In line with the alteration of DNA methylation, gene expression analysis using qPCR showed reduced mRNA expression of *Wnt5a* and increased mRNA expression of *Pik3c2a*, *Pik3c2b*, and *Pik3r2* in mice exposed to HF-inulin compared with mice in the HF group (*P* < 0.01, Figures 5B,D,F,H). Together, our results suggested that inulin supplementation might alter *Wnt5a*, *Pik3c2a*, *Pik3c2b*, and *Pik3r2* gene methylation and mRNA expression in mouse pups from HF-exposed dams.

## Discussion

In this study, we investigated the effect of maternal inulin treatment on offspring glucose metabolism at weaning. The results revealed that a maternal HF diet induced early-onset diabetes among male offspring at weaning. Maternal inulin treatment moderated glucose intolerance and insulin resistance caused by maternal HF diet exposure. Several studies had drew the similar conclusion about the benefit of inulin (Hallam and Reimer, 2014; Dennison et al., 2017). However, recent one study found that C57BL/6J mice fed a HF diet could be susceptible to liver cancer upon consumption of inulin (Singh et al., 2018). In clinical trials, it did not show adverse effects of inulin on liver function in human (Wang et al., 2019). The reason might be the duration of inulin treatment and HF diet. In Singh’s study, mice fed with HF diet and inulin for 6 months.

The “first 1000 days” concept states that the intrauterine year and the first two years of a child’s life is a critical stage of human development and health that impacts the rest of the child’s life (Berg, 2016). Numerous interventions have shown the bifidogenic effects of inulin in infants and children (Meyer and Stasse-Wolthuis, 2009; Roberfroid et al., 2010), and inulin is widely applied in infant formula today for its prebiotic properties and more recently in milk for toddlers. Inulin supplementation during the first 1000 days has an important effect on the health of the infant, such as effects related to infections and the immune system (Thorburn et al., 2015; Firmansyah et al., 2016). Moreover, the potential of inulin in metabolic programing has also been addressed. Maternal dietary inulin affects the intestinal microbiota in suckling piglets (Passlack et al., 2015). Postnatal inulin intake in offspring exposed to maternal protein restriction moderated insulin resistance in male rat offspring (Hallam and Reimer, 2014). Additionally, a maternal high-prebiotic-fiber diet could reduce the incidence of obesity induced by a HF diet in adulthood (Hallam and Reimer, 2013). Unlike the postnatal period, few studies have focused on the effect of inulin treatment *in utero* on infants. Maternal oligofructose treatment in obese female rats during gestation and lactation reduced offspring blood glucose in 17-week-old rats through the gut microbiota (Dennison et al., 2017). Metabolite production by the gut microbiome may affect epigenetic changes. Butyrate is a SCFA and is a potent inhibitor of histone deacetylases (HDACs) (Davie, 2003). Some recent research has focused on the linkage of the gut microbiota and host epigenetic modification. In a pilot study among pregnant women, the dominant abundance of Firmicutes or Bacteroidetes in the gut microbiota was associated with differential blood DNA methylation linked to lipid metabolism and obesity (Kumar et al., 2014).

In this study, maternal inulin treatment increased *Wnt5a* gene methylation expression in the livers of mice exposed to a maternal HF diet. Moreover, *Wnt5a* gene expression decreased in the inulin group. Some recent research revealed a direct effect of disturbed WNT signaling on metabolic diseases, such as insulin resistance, inflammation, and T2D (Kikuchi et al., 2012). Schulte et al. (2012) showed that serum WNT5A increased dramatically in obese subjects with low-grade inflammation via non-canonical signaling. In rats with T2DM associated with non-alcoholic steatohepatitis (NASH), *Wnt5a* mRNA and protein expression increased in the liver (Tian et al., 2014). C-Jun N-terminal kinase (JNK) is a downstream molecule in the non-canonical WNT signaling pathway (Veeman et al., 2003). Inhibition of the WNT5A/JNK1 axis improved insulin sensitivity and metabolic function (Ouchi et al., 2010). Therefore, early maternal inulin intervention may moderate insulin resistance by inhibiting *Wnt5a* in male offspring.

In the inulin group, we also observed lower methylation and higher gene expression of *Pik3c2a*, *Pik3c2b*, and *Pik3r2*, three genes involved in a phosphorylation cascade. Phosphoinositide 3-kinase (PI3K) is a key switch in the insulin signaling pathway. PI3K regulates the phosphorylation of protein kinase (PKB) and phosphoinositide-dependent protein kinase (PDK) cascades (Shepherd et al., 1998). A maternal HF diet led to PI3K regulatory subunit 1 (*Pi3kr1*), PI3K regulatory subunit 3 (*Pi3kr3*), and PI3K catalytic subunit type 2 beta (*Pi3kc2b*) hypermethylation and lower expression than that in rats fed a normal diet (Remely et al., 2014). Inulin may inhibit *Pi3k* methylation to activate *Pi3k* expression to moderate glucose metabolism.

## Conclusion

**FIGURE 6:**
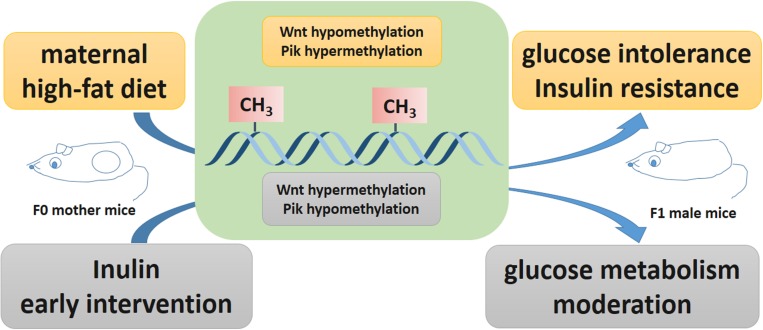
Potential effect of inulin treatment reversed epigenetic changes on gene expression affected by maternal high fat diet. Maternal high fat diet led hypomethylated *Wnt5a*, hypermethylated *Pik3c2a*, *Pik3c2b*, and *Pik3r2* in pup livers. Maternal inulin reversed these epigenetic changes in pup livers exposed to maternal high fat diet.

In summary, this paper utilized a genome-wide DNA methylation array to identify epigenetic modifications in offspring affected by maternal early inulin treatment for the first time. Maternal inulin intervention may improve glucose intolerance and insulin resistance resulting from maternal HF diet exposure by modifying DNA methylation of *Wnt5a* and *Pi3k* in the liver (Figure 6). This finding may help to identify the therapeutic target molecule to manage diabetes, especially for offspring exposed to maternal obesity and diabetes. Further work is needed to underline the detail mechanism of maternal inulin on these signaling pathway and the relationship with gut microbiome and metabolites. Moreover, the effect of maternal inulin treatment on female offspring and F2 generation glucose metabolism remain to be explored.

## Data Availability Statement

The datasets generated for this study can be found in the DNA methylation array data are available from the NCBI’s Gene Expression Omnibus repository (GEO; http://www.ncbi.nlm.nih.gov/geo/) under the series accession number GSE136766.

## Ethics Statement

All experiments were performed in accordance with the Guide for the Care and Use of Laboratory Animals, 8th ed., 2011, using protocols approved by the Animal Care Committee of Peking Union Medical Hospital (Permit Number: MC-07-6004).

## Author Contributions

XX conceived and designed the experiments and contributed reagents, materials, and analysis tools. QZ, JZ, TW, and XW performed the experiments. MY, ML, and FP analyzed the data. QZ wrote the manuscript.

## Conflict of Interest

The authors declare that the research was conducted in the absence of any commercial or financial relationships that could be construed as a potential conflict of interest.

## Footnotes

1 http://david.abcc.ncifcrf.gov/

2 http://www.ncbi.nlm.nih.gov/geo/
