# Supplementary material for: Maternal Inulin Supplementation Alters Hepatic DNA Methylation Profile and Improves Glucose Metabolism in Offspring Mice
Source: Front Physiol. 2020 Feb 7;11:70. doi: 10.3389/fphys.2020.00070 (PMC7020697; doi:10.3389/fphys.2020.00070)
Supplement: Supplementary file 1 [file Table_1.docx]

Supplemental Table 1. Significant GO term of differentially methylated genes between HF-inulin group and HF group (adjusted *P*<0.01)

| category | Term ID | Term name | gene count | Adjusted P Value | Genes |
| --- | --- | --- | --- | --- | --- |
| biological process | GO:0007275 | multicellular organism development | 66 | 0.001974 | STIL, SLC22A16, NRP1, PDGFA, PAX2, HOXD10, HOOK1, HOXC9, WNT3, CCBE1, SPRED3, OLIG1, POU4F1, ANGPT2, SCX, TWIST2, ALX1, WNT10B, NOTO, PDPN, RREB1, NKX2-6, PLXNB2, DPPA5A, ARHGAP24, MIXL1, FMN2, ARVCF, SENP1, CAMK1, SEMA4B, RIPPLY2, GADD45B, IGSF9, RAD17, TSNAXIP1, WNT5A, SHROOM2, HOXA11, NANOS2, PAQR7, T, TPI1, KRT8, FBXW4, POU3F3, ODF1, SPATA6, TMEM198, SPATA5, HELLS, WNT8B, TRNP1, GSX1, FOXA1, ISL1, GGN, NKX6-1, FEZF2, FZD10, TBATA, SFRP4, HOPX, HOXB9, C77370, CIT |
| biological process | GO:0045944 | positive regulation of transcription from RNA polymerase II promoter | 64 | 0.002153 | HNF1A, ARNT2, XRCC6, PAX2, HOXD10, WBP2, MAMSTR, GATA2, S1PR1, GATA5, PSIP1, IL1B, RARA, PATZ1, POU4F1, SCX, NR2F1, ALX1, CIITA, AR, WNT10B, ARID5A, IL25, CDK9, MED13, CD40, DDN, LPIN1, MIXL1, JUNB, ADRB2, SENP1, IFNB1, CAMK1, WNT5A, ABLIM2, PUS1, UBE3A, HDGF, ARID2, T, REL, AUTS2, NKX2-1, POU3F3, TAF9, TCEA1, ZFP292, TCEA2, NFATC2, PIK3R2, GSX1, FOXA1, KLF15, ISL1, HDAC3, DLX6OS1, CSRNP2, HOXB9, RAD54L2, IRF4, KLF2, NFIB, IL2 |
| biological process | GO:0016358 | dendrite development | 7 | 0.004409 | FEZF2, NRP1, GRIP1, GHRL, CIT, IGSF9, SCARF1 |
| biological process | GO:2000009 | negative regulation of protein localization to cell surface | 4 | 0.005787 | LEPROTL1, ACTN2, LEPROT, TAX1BP3 |
| biological process | GO:0040014 | regulation of multicellular organism growth | 7 | 0.005802 | HDAC3, HTRA2, FTO, ATRN, NPPC, SPR, PRLH |
| cellular component | GO:0016020 | membrane | 361 | 6.80E-06 | STARD3NL, QPCTL, XRCC6, LPAR1, AQP6, RNF215, MEGF11, HS2ST1, MALL, S1PR1, HTRA2, APOE, PLOD3, CH25H, PSENEN, ADAM9, AUP1, GTPBP1, TMEM150C, CRTAM, BSG, GJD3, C1GALT1C1, MAGI1, GATM, RAN, PDPN, TTC7B, CDHR5, MED13, CD40, LHFP, LPCAT2, NCAPD2, LHFPL3, WFDC8, LHFPL5, LHFPL4, PTRF, SPAG4, HTR7, DOC2G, TMEM170B, EXT1, MDH2, LY6G6D, GCNT4, CDK5R1, TMX4, MTX1, ITGA11, CACNB1, CHCHD3, XK, SFXN1, DAAM1, TMEM229B, VMN1R45, HCRTR2, SLC32A1, RILPL1, 1600002K03RIK, SYBU, ZAP70, LETMD1, PABPC1, RPS23, GUCY2G, SPNS3, SCD2, PLB1, TM2D2, ACER2, RHBDF1, TREX1, TAX1BP3, KCNK1, RPS6, KCNK2, MARCH11, CYP7B1, GNGT2, SYNE2, TREML4, HSPA14, SYTL3, UTS2R, SMURF1, ADAR, STEAP3, CYP2J5, JPH3, NRP1, LEPROTL1, SLC20A1, HIP1R, SLC20A2, SLC15A2, GRIP1, SUSD2, ATP10A, H2-D1, HELZ, LTC4S, KCNIP3, ART4, ATAD3A, NOD1, FANCI, AGPAT9, FAM129A, GOLGA5, ATP5H, JAKMIP1, ZDHHC2, GPR174, PIK3C2A, SPECC1, SLC3A2, NDUFA11, FMN2, CLIC3, CLIC4, DOK7, SEMA4B, VCAN, GLRA4, GNAS, SUSD3, TMCO1, RAB3A, SHROOM2, HSD3B1, MTDH, REN1, ABI3, HIST2H3C1, WTIP, TPCN2, MIP, NPDC1, MTMR3, GNPTAB, HPSE2, FAT2, EXOC4, 4932438A13RIK, PHLDA3, NSDHL, MARS, PRSS30, A4GALT, COX7A1, RRBP1, DLGAP2, TMEM9, ATRN, H2-AB1, TMEM5, GABARAP, PREB, GBA2, RAB30, CSNK1D, ILF2, AP2A1, LAYN, KREMEN1, LIME1, UBXN8, PTCH2, BAMBI, CIT, NCLN, UBXN4, CMTM5, KCNC2, SLC22A16, XPO1, SLC44A1, KCNC4, SLC13A5, PDGFA, SLC22A15, TIRAP, TNFSF13, ITPKB, ILDR1, VIPR1, RELL1, IQGAP1, RELL2, ELOVL1, SLC2A5, FAM49B, SLC2A4, ST3GAL4, SLC25A22, SPRED3, IL15RA, SLC4A2, RPN2, LEPROT, ANKZF1, SCAMP5, ATG9A, NBAS, PLXNB2, ERP29, SLC22A23, STAP2, SOCS7, MRGPRE, SLC22A20, PROKR1, RPH3A, BICD1, HNRNPU, CYP4X1, COPG2, CHID1, CHPF, CD300LF, CLIP1, IGSF9, SEC61G, FXYD1, SNX9, DERL2, SLC38A3, ANTXRL, BBS7, MRAP, DRD5, SNX8, LRBA, PAQR7, CTSA, ST8SIA2, UBAC2, ITGB1, RPL10L, IL12RB2, MRS2, SLC48A1, DDX46, HIST1H4A, TOMM6, RNFT2, MTCH1, OTOP2, RNFT1, TMEM198, ENTPD2, PARD6A, TBC1D10C, ADAM23, CPNE6, CKAP4, TNFRSF13B, DOCK9, GOLIM4, NIPA2, BFSP2, UCP1, ATAD1, NMT2, LAMP1, ITGB2L, LOXHD1, HIST1H3A, CACNA1G, COQ10B, RHBDL2, SYT15, YIPF4, TGTP2, TMPO, SPCS2, HIST1H3E, SCARA3, HIST1H3G, DCXR, DNAJB6, SLC25A17, COPA, CXCR1, RGP1, RHOU, SDC2, PLEKHB2, ARHGAP21, GPC5, TAAR6, SLC35D3, ZFYVE16, SLC1A7, NIPAL2, LBP, RAMP1, BOC, SLC43A2, SGK1, PARM1, HEPHL1, CDK9, SRPRB, RASL12, DDN, CDK5, LPIN1, MCM5, COQ3, EIF4G2, FAM134C, ADRB2, PSD, DGAT2, CD82, KRIT1, CHRM1, ARCN1, MFSD10, PPM1L, PDE9A, SNX10, PHLPP2, NDUFB7, SCARF1, TUBGCP3, SLC35B3, TOR1A, TOR1B, CD4, PLCD1, PIK3R5, CATSPERG1, EHD1, SCNN1A, VPS36, CRIM1, VSTM2B, TMC6, HPN, ABHD15, DNM1L, TMBIM6, TSPAN14, DCST1, MAN1C1, NCSTN, ATXN2, LMBR1L, FZD10, ITPRIPL1, PRLR, TMEM43, IRF4, GGA2, RNF40, OPRD1 |
| cellular component | GO:0005634 | nucleus | 312 | 3.74E-05 | HIST2H2AA2, TEX19.2, HMGN2, XRCC6, SNIP1, HOXD10, HIST2H2AA1, MAMSTR, TRAK2, HTRA2, APOE, FAM53A, PHTF1, RARA, PATZ1, TWIST2, MAGI1, RAN, NKX2-6, RREB1, ANAPC4, GSTT1, MED13, ESCO2, NCAPD2, STRA13, PPP1CA, RFC4, PTRF, BAZ1B, SPAG5, SMARCAL1, NLE1, CAMK1, ZNHIT1, TGIF2, MDH2, CDK5R1, PUS1, HOXA11, CHCHD3, T, TPI1, ACD, LHX4, TCTEX1D4, NKX2-1, PABPC1, LGALS1, FTO, TREX1, KLF15, RPS6, TAX1BP3, U2AF1L4, KCNK2, NOP10, RPF1, GGN, NKX6-1, LGALS9, HN1L, CBLC, SYNE2, CSRNP2, PKP3, SFRP4, HOPX, ZFP800, ZIK1, RAD54L2, SMURF1, KLF2, ZFHX3, ADAR, RANBP10, ZFP385C, ZFP46, HNF1A, HELZ, LTC4S, FOXS1, KCNIP3, SENP6, P4HA2, FANCI, PARG, DMRTC1A, FANCG, ANGPT2, FANCA, SCX, NR2F1, NFKBIZ, AR, CCNJ, RBBP4, NOTO, ANP32B, RRP36, PIK3C2A, SPECC1, SLC3A2, RAD52, MXD1, FMN2, ARVCF, IGFN1, SENP1, CLIC3, CLIC4, LYZL4, RIPPLY2, GNAS, GADD45B, CRYBA1, MTDH, HIST1H2AF, CETN2, NLRP1A, HIST2H3C1, WTIP, MIF, SNRK, RB1CC1, FAT2, TCEA1, TCEA2, 4932438A13RIK, MLLT3, RBM22, RBM24, STRADB, CAPN3, MIS12, FSD1, PREB, SUGP2, TBATA, ILF2, UBTF, CSNK1D, PHF13, OBFC1, HSPA4L, UBXN4, XPO1, XPO6, GNPDA2, STK35, ARNT2, SNRPD2, ITPKB, ZFP787, ZFP580, IQGAP1, GATA2, EIF4EBP1, HOXC9, GATA5, EED, PSIP1, SRRM1, IL15RA, PITPNC1, OLIG1, RPN2, ZFP276, CIITA, NUDT6, TRIM41, SOCS7, XRRA1, HNRNPU, JUNB, TACC2, CHID1, MAD2L1BP, KDM2B, HSPB6, SNRPB, DCUN1D4, RAD17, BBS7, SSH3, HDGF, TRRAP, RBX1, RPL10L, IRAK3, POLE4, DDX46, HIST1H4A, CNOT6L, AUTS2, NPM3, RNF11, TAF9, UNKL, HELLS, PARD6A, MAFF, TRIP4, FOXA1, GSX1, SUGT1, FOXP4, ARMC1, ATAD1, SALL3, FEZF2, DLX6OS1, HDAC3, HIST1H3A, RFX1, HOXB9, TMPO, HIST1H3E, ABL1, HIST1H3G, DCXR, DNAJB6, MYH10, KIF23, E2F2, RSF1, PRDX4, DPH3, PAX2, PAK6, MT2, RANBP1, HIST3H2A, POU4F1, FAM83G, TUBA1C, NFX1, ALX1, SGK1, PARM1, DDB1, ARID5A, CDK9, ZFP28, DDN, CDK5, LPIN1, MCM5, MIXL1, GZF1, RFWD2, CD3EAP, ADRB2, ZPBP2, FAM120B, UBE2K, SERPINA3G, HORMAD1, 1110004E09RIK, UBE2S, CPSF1, UGP2, SNX10, WIZ, PRKCZ, BCLAF1, PHLPP2, IRX5, HYLS1, UBE3A, NANOS2, IRX2, TRA2A, ADH5, SMUG1, STUB1, RPA3, PRDM12, REL, TOR1A, KRT8, TOR1B, GTF3C5, POU3F3, NEFH, PLCD1, PIK3R5, HSPA4, ODF1, ZFP292, NFATC2, VPS36, PIK3R2, TERF1, MSH6, NACC2, TRNP1, UBE4B, BMYC, RCAN1, SIRT7, ISL1, UBE2Q1, RALGDS, KRT73, TOX, ZFP182, TMEM43, IRF4, C77370, RNF40, NFIB |
| cellular component | GO:0005737 | cytoplasm | 328 | 8.33E-04 | TEX19.2, HMGN2, PLEKHM2, CROCC, THOP1, XRCC6, SNIP1, LPAR1, HOOK1, WDR72, TRAK2, APOD, STAC, APOE, PHTF1, RARA, DHX35, PRLH, TWIST2, ADAM9, AUP1, GTPBP1, MAGI1, RAN, RREB1, TTC7B, GSTT1, DPPA5A, CD40, NCAPD2, PPP1CA, PTRF, SPAG5, SPAG4, CAMK1, KIF26A, CDK5R1, CHCHD3, UBA6, AFAP1L2, DAAM1, PXN, T, RILPL1, ACD, CRMP1, 1600002K03RIK, ZAP70, KBTBD8, TCTEX1D4, PABPC1, RPS23, UNC119, CRIP1, LGALS1, TREX1, KCNK1, RPS6, TAX1BP3, U2AF1L4, LGALS9, GGN, HN1L, CCT4, SYNE2, RNF208, PTAR1, SFRP4, HOPX, HSPA14, SMURF1, ZFHX3, ADAR, RANBP10, STEAP3, ZFP46, HNF1A, HIP1R, TTLL9, RASSF7, KCNIP3, SENP6, NOD1, TMSB15A, P4HA2, CEP250, FANCI, PARG, LMOD1, FANCG, FBXL15, FAM129A, FANCA, ATP5H, JAKMIP1, ARHGEF3, EIF2S3X, AR, ANP32B, PIK3C2A, SLC3A2, ARHGEF16, GMPS, FMN2, ARVCF, MYRIP, IGFN1, SENP1, CLIC3, ADM, CLIC4, IL12A, GNAS, GADD45B, CRYBA1, MON1B, TSNAXIP1, SHROOM1, PPFIA3, SHROOM2, MTDH, REN1, ABI3, CETN2, NLRP1A, LIX1, SERPINB1B, WTIP, MIF, SPHKAP, MTMR3, DNALI1, SNRK, RB1CC1, AGT, EXOC4, PCMT1, USP33, SPATA5, PHLDA3, MARS, RBM22, RBM24, ATRN, LRRC45, STRADB, CAPN3, GABARAP, FSD1, TBATA, PPP1R2, RAB30, CSNK1D, ILF2, HSPA4L, PSAT1, CIT, BAMBI, UBXN4, STIL, CEP72, XPO1, GNPDA1, XPO6, GNPDA2, STK35, ARNT2, TIRAP, IQGAP3, SNRPD2, TNFSF13, IQGAP1, TGFB2, EIF4EBP1, NLRC3, WNT3, SLC2A4, IL1B, PITPNC1, SPR, TUBG1, VMAC, ATG9A, NUDT6, STAP2, TRIM41, HAL, SOCS7, ACTN2, XRRA1, HNRNPU, TACC2, COPG2, MAD2L1BP, HSPB6, CHPF, SNRPB, CLIP1, NEURL2, SEPT9, TRAF1, WNT5A, SNX9, ARPP21, BBS7, SSH3, HDGF, UBAC1, ITGB1, RBX1, IRAK3, EIF3B, CNOT6L, ANKIB1, RNF11, UNKL, RHOBTB3, PARD6A, GM853, TRIP4, CKAP4, CPNE6, RPGRIP1L, BFSP2, KCTD2, SUGT1, NMT2, LAMP1, HDAC3, CACNA1G, ABL1, DNAJB6, MYH10, SLC25A17, KIF23, COPA, PODNL1, PRDX4, RGP1, DPH3, NDUFAF1, PAK6, ARHGAP21, TRIM3, SLK, ZFYVE16, MT2, RANBP1, KLHL24, PLCB2, LOXL1, FAM83G, TUBA1C, NFX1, SGK1, PARM1, DDB1, CDK9, PFKM, SRPRB, ARHGAP24, DDN, CDK5, LPIN1, GZF1, RFWD2, CD3EAP, EIF4G2, TARS, ADRB2, SERPINA3G, ATG4B, UBE2K, KRIT1, ARCN1, GHRL, LCE3F, PDE9A, HORMAD1, 1110004E09RIK, UBE2S, UGP2, SNX10, CASQ2, ABLIM2, PRKCZ, BCLAF1, PHLPP2, HYLS1, UBE3A, NANOS2, VIM, ADH5, STUB1, SERPINB9C, TUBGCP3, REL, TOR1A, KRT8, SEC16A, TOR1B, NEFH, PLCD1, PIK3R5, HSPA4, NFATC2, VPS36, TERF1, MSH6, TMC6, HPN, DNM1L, GIMAP8, TMBIM6, UBE4B, GRSF1, DPYSL5, RCAN1, SIRT7, UBE2Q1, RALGDS, ATXN2, FZD10, MPI, IRF4, APIP, ARAP2, OPRD1 |
| cellular component | GO:0005667 | transcription factor complex | 24 | 0.0012 | E2F2, TRIP4, HNF1A, PUS1, HOXA11, XRCC6, ARNT2, ARID5A, TRRAP, WTIP, PAX2, LPIN1, ARID2, JUNB, GATA2, GATA5, REL, NKX2-1, OLIG1, NFATC2, ZFHX3, SCX, TWIST2, ALX1 |
| cellular component | GO:0000139 | Golgi membrane | 31 | 0.002438 | GCNT4, COPA, QPCTL, RGP1, RHOU, HS2ST1, ARHGAP21, MALL, GNPTAB, GALNTL6, SEC16A, SYBU, LEPROT, GOLGA5, SCAMP5, PARM1, A4GALT, DNM1L, RHBDF1, TMEM5, LPCAT2, MAN1C1, GABARAP, RFWD2, PREB, GBA2, COPG2, ARCN1, SCARA3, EXT1, TMCO1 |
| molecular function | GO:0005515 | protein binding | 233 | 6.56E-06 | TEX19.2, CROCC, XRCC6, SNIP1, HOOK1, MAMSTR, APOE, GIT2, RARA, PSENEN, PATZ1, HTRA3, TWIST2, CUL1, ADAM9, MAGI1, RAN, CD40, LHFPL5, PPP1CA, BAZ1B, F2, CAMK1, KIF26A, CDK5R1, PUS1, A, HOXA11, CHCHD3, CACNB1, UBA6, CHCHD4, PXN, HCRTR2, T, ACD, ZAP70, NKX2-1, PABPC1, UNC119, CRIP2, LGALS1, TREX1, KLF15, HS1BP3, KCNK1, RPS6, TAX1BP3, U2AF1L4, NOP10, KCNK2, GGN, CCT4, PKP3, HOPX, TREML4, RAD54L2, TRAFD1, SYTL3, SMURF1, KLF2, ADAR, STEAP3, NRP1, HNF1A, HIP1R, GRIP1, H2-D1, HELZ, WBP2, NOD1, P4HA2, ANGPT2, SCX, AR, RBBP4, PIK3C2B, SLC3A2, MXD1, GPR179, ARVCF, MYRIP, SENP1, DOK7, SEMA4B, GNAS, GADD45B, TSNAXIP1, RAB3A, SHROOM2, MTDH, NLRP1A, WTIP, MIP, RB1CC1, EXOC4, TCEA1, USP33, MLLT3, RRBP1, ATRN, H2-AB1, CAPN3, GABARAP, RASSF6, TBATA, RAB37, UBTF, CSNK1D, AP2A1, OBFC1, HSPA4L, LIME1, CIT, KCNC2, XPO1, STIL, TIRAP, IQGAP3, IQGAP1, TGFB2, GATA2, EIF4EBP1, NLRC3, WNT3, FAM49B, GATA5, SLC2A4, SEP15, EED, LEPROT, TUBG1, PLXNB2, STAP2, TRIM41, SOCS7, ACTN2, RPH3A, JUNB, HNRNPU, MAD2L1BP, SNRPB, CLIP1, CD300LF, NEURL2, TRAF1, WNT5A, SNX9, HDGF, CTSA, ITGB1, RBX1, IL12RB2, IRAK3, CNOT6L, AUTS2, NPM3, RNF11, ENTPD2, RHOBTB3, PARD6A, ADAM23, CPNE6, NIPA2, BFSP2, UCP1, ATAD1, LAMP1, HDAC3, DLX6OS1, CACNA1G, TMPO, ABL1, DCXR, DNAJB6, MYH10, E2F2, PAX2, SDC2, TRIM3, SLK, SLC35D3, POU4F1, LOXL1, BOC, NFX1, ALX1, SGK1, SLC7A15, CDK9, PFKM, LPIN1, CDK5, RFWD2, CD3EAP, EIF4G2, ADRB2, PPM1J, FAM120B, ATG4B, KRIT1, PPM1L, HORMAD1, WIZ, PRKCZ, BCLAF1, UBE3A, NANOS2, VIM, STUB1, SCARF1, REL, KRT8, TOR1A, ACAN, TOR1B, POU3F3, HSPA4, PLCD1, NFATC2, EHD1, PIK3R2, TERF1, TRNP1, UBE4B, DPYSL5, BMYC, ISL1, RALGDS, NCSTN, PRLR, IRF4, RNF40 |
| molecular function | GO:0003677 | DNA binding | 116 | 7.46E-05 | HIST2H2AA2, HMGN2, ARNT2, XRCC6, ZFP787, HOXD10, ZFP580, HIST2H2AA1, GATA2, HOXC9, GATA5, PHTF1, PSIP1, SRRM1, RARA, OLIG1, PATZ1, ZFP276, TWIST2, NKX2-6, RREB1, JUNB, HNRNPU, STRA13, KDM2B, RFC4, TGIF2, HOXA11, SSH3, ZFP560, HDGF, T, POLE4, ACD, HIST1H4A, LHX4, NKX2-1, TAF9, MAFF, GSX1, FOXA1, KLF15, FOXP4, NKX6-1, SALL3, FEZF2, HDAC3, CSRNP2, RFX1, HOPX, ZFP800, HOXB9, RAD54L2, ZIK1, TMPO, HIST1H3E, KLF2, ABL1, ZFHX3, DNAJB6, AHDC1, ADAR, E2F2, HNF1A, ZFP46, PAX2, KCNIP3, FOXS1, POU4F1, HIST3H2A, SCX, NFX1, NR2F1, ALX1, AR, NOTO, HIST3H2BB-PS, DDB1, ARID5A, CDK9, MXD1, RAD52, ZFP28, MCM5, MIXL1, GZF1, BCLAF1, IRX5, HIST1H2AF, THAP4, THAP3, IRX2, HIST2H3C1, ARID2, SMUG1, RPA3, PRDM12, REL, GTF3C5, POU3F3, TCEA1, ZFP292, TCEA2, NFATC2, TERF1, MSH6, TRNP1, ISL1, PREB, TOX, ILF2, UBTF, ZFP182, OBFC1, IRF4, NFIB |
| molecular function | GO:0017048 | Rho GTPase binding | 8 | 8.51E-04 | PARD6A, ARHGEF16, IQGAP3, DOCK9, RTKN, DAAM1, CIT, IQGAP1 |
| molecular function | GO:0019904 | protein domain specific binding | 24 | 0.004667 | WNT5A, PRKCZ, XPO1, SHROOM2, AR, DLGAP2, RAN, FOXA1, ACTN2, CD40, ITGB1, IQGAP1, TACC2, LAMP1, WNT3, PPP1R2, HIST1H4A, CEP250, IL1B, GNAS, RARA, ODF1, ABL1, TWIST2 |
| molecular function | GO:0004722 | protein serine/threonine phosphatase activity | 8 | 0.008242 | PDP1, MTMR3, PPP1CA, PHLPP2, PPM1J, PPM1K, PPM1L, PPP2R2C |

Supplemental Table 2. Significant KEGG pathway of differentially methylated genes between HF-inulin group and HF group (adjusted *P*<0.05)

| Term ID | Term name | gene count | Adjusted P value | Genes |
| --- | --- | --- | --- | --- |
| mmu05166 | HTLV-I infection | 24 | 0.001828 | WNT5A, XPO1, E2F2, WNT10B, NRP1, RAN, PDGFA, ANAPC4, H2-D1, H2-AB1, TRRAP, CD40, TGFB2, ITGB2L, FZD10, POLE4, WNT3, IL15RA, RANBP1, PIK3R5, NFATC2, IL2, PIK3R2, WNT8B |
| mmu05205 | Proteoglycans in cancer | 19 | 0.002883 | WNT5A, WNT10B, RPS6, ITGB1, HOXD10, SDC2, PXN, IQGAP1, TGFB2, EIF4B, CBLC, FZD10, PPP1CA, WNT3, HPSE2, PIK3R5, TWIST2, WNT8B, PIK3R2 |
| mmu05322 | Systemic lupus erythematosus | 15 | 0.004365 | HIST2H2AA2, HIST1H2AF, HIST3H2BB-PS, HIST2H3C1, H2-AB1, ACTN2, CD40, HIST2H2AA1, C1QB, HIST1H4A, HIST1H3A, SNRPB, HIST3H2A, HIST1H3E, HIST1H3G |
| mmu04120 | Ubiquitin mediated proteolysis | 14 | 0.008198 | RFWD2, CBLC, UBE3A, UBE2K, DDB1, ANAPC4, UBE4B, UBA6, SMURF1, STUB1, UBE2S, UBE2Q1, CUL1, RBX1 |
| mmu05146 | Amoebiasis | 12 | 0.011986 | SERPINB9C, ITGB2L, LAMB2, IL12A, IL1B, GNAS, PIK3R5, ACTN2, SERPINB1B, PLCB2, TGFB2, PIK3R2 |
| mmu05142 | Chagas disease (American trypanosomiasis) | 11 | 0.013148 | C1QB, IFNB1, IL12A, IL1B, GNAS, PIK3R5, PLCB2, PPP2R2C, TGFB2, IL2, PIK3R2 |
| mmu04390 | Hippo signaling pathway | 14 | 0.013357 | WNT5A, PARD6A, PRKCZ, WNT10B, WTIP, TGFB2, FZD10, PPP1CA, ITGB2L, WNT3, RASSF6, CSNK1D, PPP2R2C, WNT8B |
| mmu05144 | Malaria | 7 | 0.016445 | ITGB2L, IL12A, IL1B, CD40, SDC2, THBS3, TGFB2 |
| mmu05200 | Pathways in cancer | 27 | 0.020353 | TRAF1, WNT5A, FGF5, E2F2, PDGFA, ARNT2, LPAR1, ITGB1, TGFB2, RBX1, LAMB2, WNT3, RARA, PIK3R5, PLCB2, WNT8B, PIK3R2, MSH6, AR, WNT10B, RALGDS, CBLC, GNGT2, FZD10, GNAS, PTCH2, ABL1 |
| mmu04810 | Regulation of actin cytoskeleton | 17 | 0.021742 | FGF5, PDGFA, SSH3, ITGA11, IQGAP3, ACTN2, ITGB1, IQGAP1, PXN, MYL9, PAK6, ITGB2L, PPP1CA, CHRM1, F2, PIK3R5, PIK3R2 |
| mmu04672 | Intestinal immune network for IgA production | 6 | 0.033417 | TNFRSF13B, IL15RA, TNFSF13, H2-AB1, CD40, IL2 |
| mmu03420 | Nucleotide excision repair | 6 | 0.039811 | POLE4, RFC4, DDB1, CETN2, RPA3, RBX1 |
| mmu04310 | WNT signaling pathway | 12 | 0.041058 | WNT5A, FZD10, WNT10B, WNT3, SFRP4, NFATC2, DAAM1, BAMBI, PLCB2, CUL1, WNT8B, RBX1 |
| mmu04630 | Jak-STAT signaling pathway | 12 | 0.048622 | IL12RB2, PRLR, GM13305, IFNB1, GM2002, IL12A, IL11RA2, IL15RA, SOCS7, PIK3R5, IL2, PIK3R2 |
